# Discovery of the oldest South American fossil lizard illustrates the cosmopolitanism of early South American squamates

**DOI:** 10.1038/s42003-020-0926-0

**Published:** 2020-04-29

**Authors:** Jonathas Souza Bittencourt, Tiago Rodrigues Simões, Michael Wayne Caldwell, Max Cardoso Langer

**Affiliations:** 1 Departamento de Geologia, Laboratório de Paleontologia e Macroevolução (CPMTC-IGC), Universidade Federal de Minas Gerais, Belo Horizonte-MG, 31270-901 Brazil; 2 Department of Organismic and Evolutionary Biology, Museum of Comparative Zoology, Harvard University, Cambridge, MA 02138 USA; 3 Department of Biological Sciences, and Department of Earth and Atmospheric Sciences, University of Alberta, Edmonton, AB Canada T6G 2E9; 4 Departamento de Biologia, Laboratório de Paleontologia, FFCLRP, Universidade de São Paulo, Ribeirão Preto-SP, 14040-901 Brazil

**Keywords:** Palaeontology, Herpetology

## Abstract

Squamates have an extremely long evolutionary history with a fossil record that extends into the Middle Triassic. However, most of our knowledge of their early evolutionary history is derived from Laurasian records. Therefore, fundamental questions regarding the early evolution of squamates in the Southern Hemisphere, such as the origins of the extremely diverse and endemic South American fauna, remain unanswered. Here, we describe a new lizard species that represents the oldest fossil squamate from South America, demonstrating that squamates were present on that continent at least 20 million years earlier than previously recorded. The new species represents the first occurrence of the extinct squamate family Paramacellodidae in South America and displays an unusual limb morphology. Finally, our findings suggest early South American squamates were part of a much broader distribution of their respective clades, in sharp contrast to the high levels of endemicity characteristic of modern faunas.

## Abstract (web summary)

Jonathas Bittencourt et al. report the discovery of a fossil lizard in South America dated to at least 130 mya, about 20 million years earlier than previously thought. This finding suggests early lizards in South America were much more broadly distributed than extant species in this region.

## Introduction

South America is one of the world’s largest hotspots of vertebrate diversity, housing ca. 2000 species of lizards and snakes (squamates), many of which display high levels of endemicity^1,2^. This diverse herpetofauna has been estimated to have its origins back in the Cretaceous, with the earliest squamate fossil occurrences known from the Aptian/Albian (ca. 113 million years ago, Mya) Crato Formation, in northeast Brazil, represented by three lizard species of relatively controversial phylogenetic affinities^3–6^. This record expands slightly later in the Cretaceous, with additional specimens coming mostly from Argentina and Brazil. Those later records include three species of lizards known from southeastern Brazil, as well as several species of snakes known from various localities in Brazil and Argentina^6–10^. Finally, mosasaurs are known from marine deposits from Brazil, Argentina, Chile, Peru, Venezuela, and Colombia^11^. Some of those findings have revealed, for instance, important new patterns on the biogeography of early iguanians in Gondwana^10^. However, despite the increasing knowledge on the evolution and fossil record of South American squamates in the past decade^12^, there is still a great discrepancy in our understanding of squamate evolution between Gondwana and Laurasian continents during the Mesozoic. For instance, the number of known lizard species (terrestrial limbed squamates) in Laurasia is an order of magnitude higher than in Gondwana, with only ca. 10 valid fossil species of terrestrial lizards known from the latter^10,13^.

This relatively poor understanding of the Mesozoic fossil record of South American squamates creates numerous questions surrounding the early evolution of the group, including the time of the first appearance of squamates in that region, their early diversity, and how endemic those faunas might already have been during the Cretaceous. Considering the inferred divergence time (Permian-Triassic) and first appearance of squamates in the fossil record (Middle Triassic)^14^, as well as the first appearance of other lepidosaurs in South America (Late Triassic)^15^, some level of provincialism could be expected for squamate faunas by the Cretaceous (100 Myr or more after their origin). Additionally, this long temporal gap between the origin of squamates and the earliest South American records is intriguing, and squamates could in fact have appeared in South America long before the Aptian/Albian records. An improved fossil record of South American squamates can provide critical clues to understanding broader-scale squamate evolution, which to date has been based on data gathered mostly from North America, Europe, and East Asia^16–18^.

Here, we shed light on some of the above questions based on new materials from southeastern Brazil, recognized here as the remains of a new lizard species that is the oldest South American squamate known to date. This new record indicates the presence of squamates in that continent almost 20 million years earlier than previously recognized. Importantly, this new material provides evidence of a family of lizards that, until now, was unknown to have ever inhabited South America. Finally, along with other recent discoveries, this new record reveals important clues about the early biogeographic history of squamates from South America.

## Results

### Geological setting

**Fig. 1:**
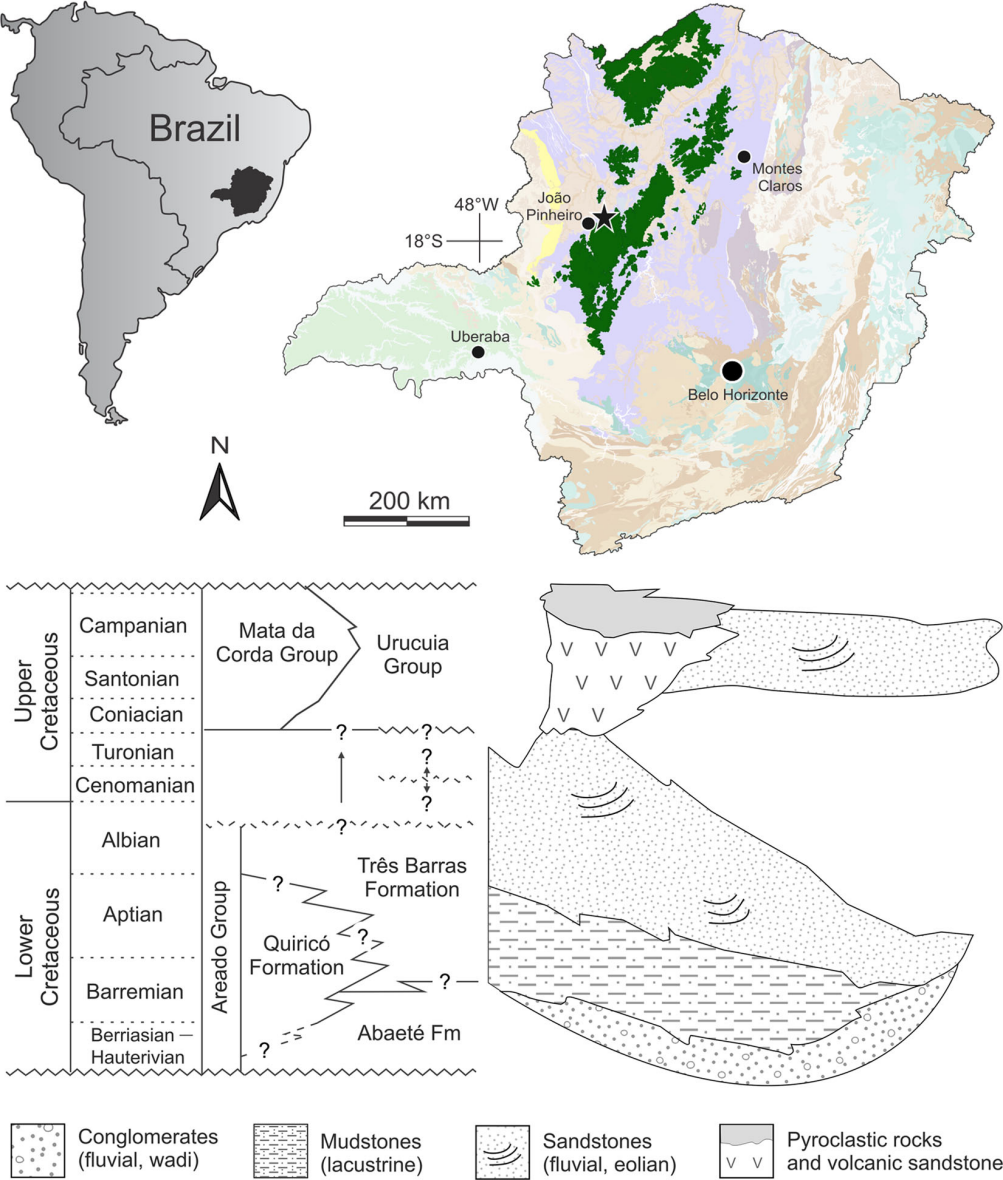
Locality and stratigraphic level of *Neokotus sanfranciscanus*. Modified from refs. ^19,70,71^.

The Quiricó Formation corresponds to the middle part of the Areado Group within the Sanfranciscana Basin^19,20^ (Fig. 1). This basin encloses most Phanerozoic rocks exposed over the São Francisco Craton, which are in a discontinuous contact with the Precambrian basement^19^ (Fig. 1). The basal Cretaceous sections (Areado Group) originated from distension forces that opened the Atlantic Ocean, creating subsidence areas within the São Francisco Cratonic realm^20^.

The beds where the specimen described here was found are composed of bioturbated mudstones, deposited in shallow lacustrine environments;^19^ these beds also yielded the coelacanthiform *Mawsonia gigas*, amiid scales and vertebrae, semionotiform scales, hybodontiform cephalic spines, along with ostracod and conchostracan carapaces (see ref. ^21^, and references therein). The fossil record of the Quiricó Formation as a whole also includes terrestrial palynomorphs, terrestrial and aquatic plants (conifers and angiosperms), arthropods (insects and crustaceans), other actinopterygians, and dinosaurs^21^.

The middle to upper layers of the Quiricó Formation correspond to a Barremian to early Aptian interval, as evidenced by the presence of ostracods, including *Wolburgiopsis plastica* and *W. chinamuertensis*, and the palynomorphs *Tucanopollis crisopolensis* and *T*. *annulatus*^22–24^. A recent study reported carapaces of the podocopidan ostracod *Cypridea hystrix*, in association with *Penthesilenula martinsi*, *Cypridea conjugata*, *Cypridea jequiensis*, *Neuquenocypris (Protoneuquenocypris) antiqua*, and *Timiriasevia sanfranciscanensis*, in the lower levels of the Quiricó Formation, which is the same of the specimen described herein, assigning a Valanginian age for this horizon^24^. Albeit such assignment still requires corroboration with additional data, the available evidence indicates that the lower portion of the Quiricó Formation, and therefore the new taxon described here, is ca. 132.6–139.8 million years old^24,25^.

### Systematic Paleontology

Squamata Oppel, 1811

Paramacellodidae Estes, 1983

*Neokotus sanfranciscanus* gen. et sp. nov.

**Holotype** The holotype is housed in the publicly available paleontological collection of the Instituto de Geociências, Universidade Federal de Minas Gerais (IGC-P), in Belo Horizonte-MG, Brazil. All the osteological remains representing the holotype of the new species (IGC-P 0085) were found in a single small (60 g) mudstone block. They are here attributed to a single individual because all identifiable bones came from a single block, all elements from the block have squamate features, and there is no duplication of elements. Additionally, the lower jaw dentition (highly diagnostic for lizards) matches that of the maxilla and premaxilla. Further, all skull elements are of the expected size for a single individual, postcranial elements of the same kind (vertebrae) are of similar size, and have proportional sizes relative to the cranial bones. The elements recovered include: skull bones, including premaxilla, maxilla, jugal, articulated dentary and splenial; remains of at least 20 vertebrae, including four dorsal, two sacral, and eleven caudal elements (other vertebrae are unidentified); rib fragments; possible fragment of the distal portion of the left humerus; incomplete radius and ulna; incomplete and articulated left ilium, pubis and ischium; right tibia; indeterminate non-terminal phalanges and unguals.

**Type locality and horizon** Quiricó Formation (Areado Group, Sanfranciscana Basin); municipality of João Pinheiro (Minas Gerais, Southeast Brazil, Fig. 1); Early Cretaceous, Valanginian (ca. 132.6–139.8 million years ago)^24,25^.

**Etymology** From ancient Greek *νεόκοτος*, new and strange, and unheard of^26^. The specific epithet alludes to the Sanfranciscana Basin, which is eponymous of the São Francisco river.

**Diagnosis** *Neokotus sanfranciscanus* can be distinguished from all other squamate species by the following combination of features: premaxillae fused to one another, dentary crista dentalis robust anteriorly and smoothly decreasing in height posteriorly*; posterior part of the dentary dorsoventrally short and bearing a narrow dental sulcus; dentary ventral crest very robust at the posterior end of the Meckelian canal*; splenial anteriorly notched and short, with its anterior end at the level of the posterior half of the dentary*; conical and relatively narrow teeth in medial view; labiolingually expanded tooth bases, with no anterolingual rotation; lingually concave tooth crowns with no accessory lingual cusps; dorsal vertebrae with a strongly developed midventral crest; ungual phalanges with a flat and laterally expanded base*. Features highlighted with an asterisk (*) are unique to this taxon among paramacellodids. In particular, the flat and laterally expanded bases of the unguals are unique among all squamates (living or extinct).

**Fig. 2:**
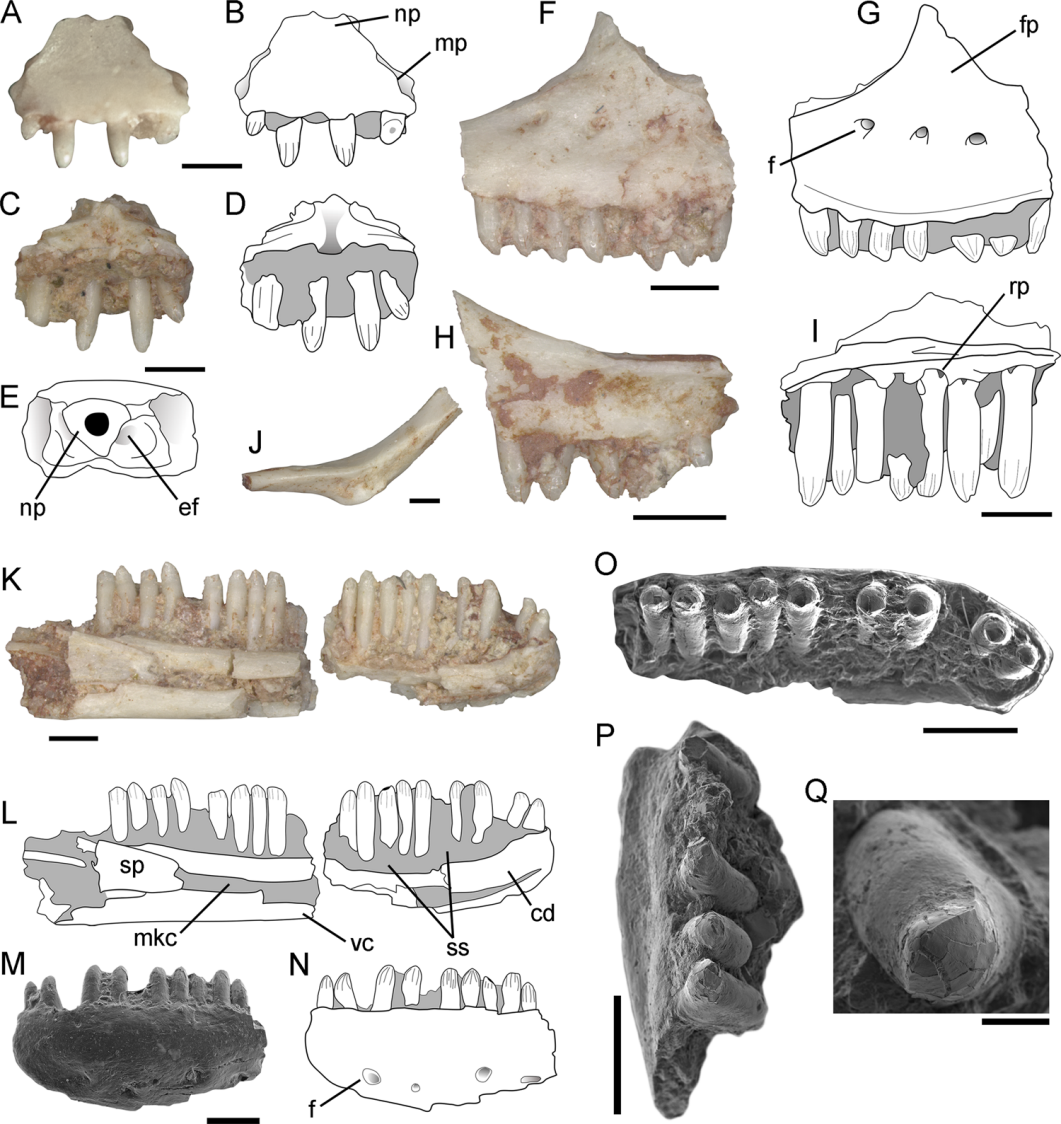
*Neokotus sanfranciscanus* holotype (IGC-P 0085). Premaxillae in (**a**–**b**) anterior, (**c**–**d**) posterior and (**e**) dorsal views. Maxillae in (**f**–**g**) lateral (left ramus), (**h**) lateral (right ramus), (**I**) medial (right ramus) views. **j** Right jugal in dorsomedial view. Left mandible (dentary and splenial) in (**k**–**l**) medial and (**m**–**n**) lateral (anterior part) views. **o** Left dentary anterior teeth. **p** Left maxillary teeth. **q** Isolated maxillary teeth. cd, crista dentalis; ef, ethmoid foramen; f, foramen; fp, facial process; mkc, Meckelian canal; mp, maxillary process; np, nasal process; rp, resorption pit; sp, splenial; ss, subdental shelf; vc, ventral crest of dentary. Scale bars: **a**–**p** = 500 µm; **q** = 50 µm.

**Morphological description** The premaxilla is medially fused to its antimere and bears a short maxillary process (Fig. 2a–e). Its anterior surface lacks foramina, with the ethmoidal foramina opening dorsocaudally through a lateral notch. The premaxilla preserves four uncuspid and unserrated teeth that are well-separated from each other and are similar in size to the anterior dentary teeth. The maxilla bears a line of mental foramina close to the base of the nasal process (Fig. 2f–i). The anterior margin of the facial process is nearly vertical and pierced by small mental foramina where it merges with the premaxillary process, on the ventral side of the external naris. The incomplete jugal is slender with a smooth outer surface (Fig. 2j).

The left dentary (Fig. 2k–o) is articulated with the anterior portion of the splenial. It is anteriorly rounded, laterally bulged, and ventrolaterally pierced by at least four mental foramina that are placed near the ventral margin of the bone. The Meckelian canal is medially open in its posterior portion, and medioventrally at its anteriormost portion. That canal is partially closed by the folded up lower border of the dentary, which creates a robust ventral crest. The subdental shelf, which floors the tooth-bearing groove, is moderately developed and inclined dorsally at the anterior end of the bone. The medial margin of the subdental shelf bears a crista dentalis that is strongly developed on the anterior third of the dentary, smoothly decreasing in robustness on the posterior two thirds. The splenial is extremely short in comparison to most squamates, including other paramacellodids (e.g., Table 1), being restricted to the posterior half of the dentary. It is notched anteriorly and does not bear any degree of fusion to the dentary.

**Table 1:** Comparative diagnostic table between paramacellodid squamates.

	Dentition lábio-lingually expanded	Dentition with lingually, concave crowns	Dentition with posteriorly curved crowns	Dentition, tooth shape on lingual view	Dentition with accessory lingual cuspule	Dentition striated lingually	Dentition, anterolingual rotation	Dentary, dorsoventral depth posteriorly	Dentary, width of dental sulcus	Dentary, crista dentalis robust anteriorly	Dentary, crista dentalis sharply decreasing in height posteriorly	Dentary, thickness of ventral crest at posterior end of Meckelian canal	Splenial, anterior extension relative to the dentary	Maxilla, tall nasal process	Dorsal vertebrae, midventral crest develop-ment
*Neokotus sanfranciscanus*	Present	Present	Absent	Conical (narrow)	Absent	?	Absent	Short	Narrow	Present	**Absent**	**Robust**	**Posterior**	?	Strong
*Paramacellodus oweni*	Present	Present	Absent	Conical (narrow)	Absent	Present	Absent	Short	Narrow	Present	Present	Thin	Anterior	Present	Mild
*Paramacellodus sinuosus*	Present	Present	Absent	Conical (acute apex)	Absent	Present	Present	Short	?	Present	Absent	Thin	?	?	?
*Paramacellodus marocensis*	Present	Present	Absent	Conical (narrow)	Absent	Present	Absent	Short	?	Present	Present	Thin	Posterior?	?	?
*Paramacellodus keebleri*	Present	Present (mild)	Absent	Chisel -shaped	Absent	Present	Absent	?	?	?	?	?	?	Present	?
*Pseudosaurillus*	Present	Present (mild)	Absent	Conical (narrow)	Absent	Present	Absent	Short	Wide	Present	Present	Thin	Anterior	Absent	?
*Saurillus obtusus*	Present	?	Absent	Conical (narrow)	Absent	Present	Absent	Short	Narrow	Present	Present	Robust	?	?	?
*Saurillodon*	Present	Present (mild)	Present	Conical (wide)	Absent	Absent	?	Deep	Narrow	Present	?	Thin	?	?	?
*Becklesius hoffstetteri*	Present	Present (mild)	Absent	Chisel -shaped	Present	Present	Absent	Short	Wide	Present	Present	Thin	Mid-length	Present	?
*Becklesius cataphractus*	Present	Present (mild)	Absent	Chisel -shaped	Present	Present	Absent	Short	?	Present	?	Thin	Mid-length	Present	?
*Atokasaurus*	Present	Present	Absent	Chisel -shaped	Present	Absent	Absent	?	Wide	Absent?	?	Thin	?	?	?

Combination of features highlighted in bold are exclusive to *Neokotus sanfranciscanus*.

The premaxillary, maxillary, and dentary teeth (Fig. 2f–i, k–q) are attached lingually to those bones, do not have their crowns ankylosed to the apex of the jaw, and are supported at their bases by a subdental shelf on the dentary and a supradental shelf on the premaxilla and maxilla. The teeth bear a conspicuous labiolingual expansion at their bases, being relatively narrow in lingual view. Their crown apices are conical and relatively straight in lingual view, bearing a moderately developed lingual concavity. The external morphology of the enamel gives the impression of crown striations on both lingual and labial sides of the teeth, but scanning electron microscopy (SEM) (Fig. 2) indicates that, at least on the labial side, those striated marks represent a fragmentation of the enamel, not true ornamentation. On the lingual side, there is also some degree of enamel fragmentation, so that it is not possible to tell if the enamel was actually striated in *Neokotus sanfranciscanus*. The crown apices do not possess any accessory cusps and the dentition is homodont on both lower and upper dental arcades. The dentary has 21 tooth positions with 17 teeth preserved in situ. Subtle resorption pits are observed on the lingual margin of the maxillary teeth, but no replacement teeth are seen.

**Fig. 3:**
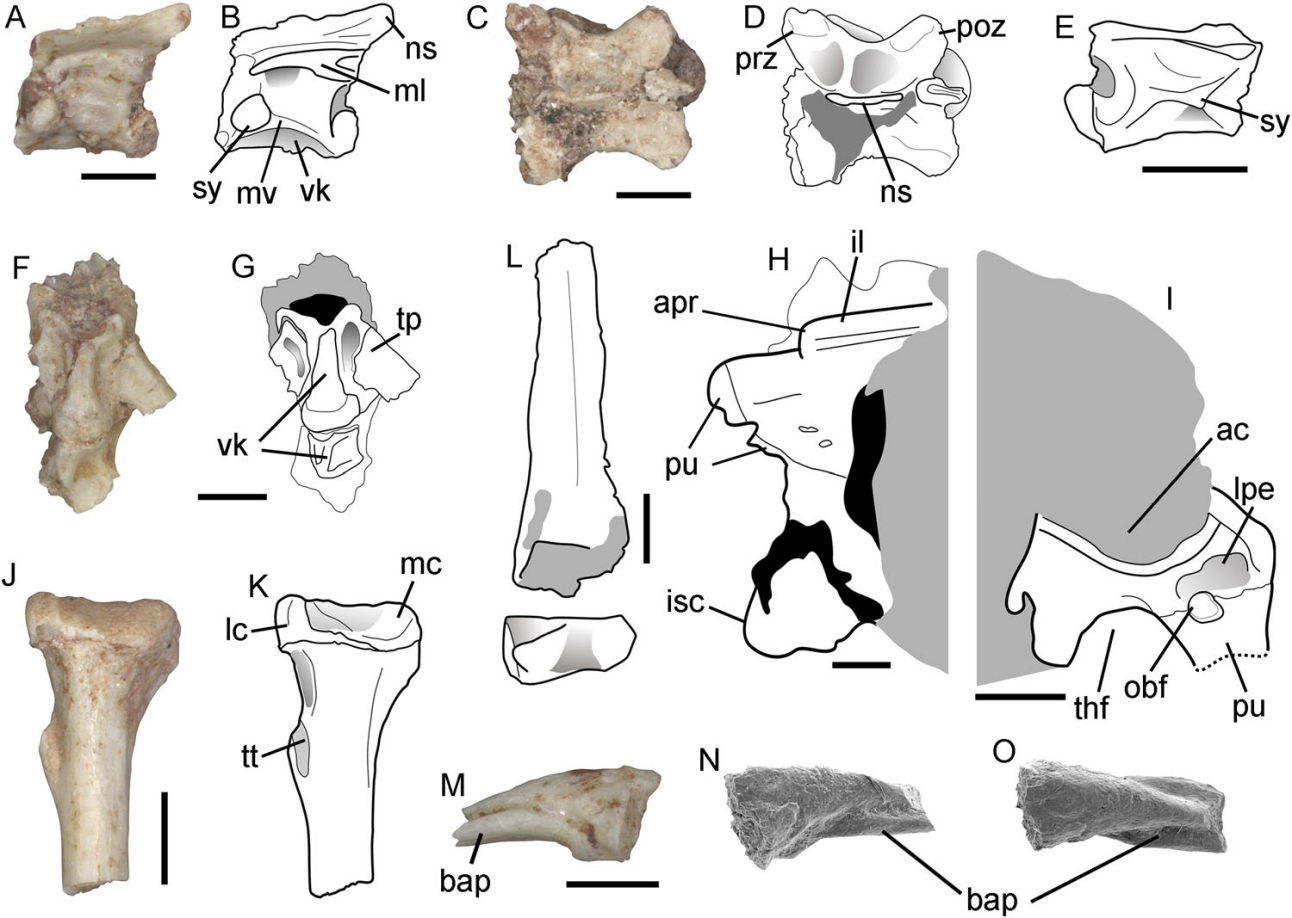
*Neokotus sanfranciscanus* holotype (IGC-P 0085). **a**–**d** Mid-dorsal vertebra in left lateral view (**a**–**b**) and in dorsal view (**c**–**d**). **e** Isolated dorsal vertebra in right lateral view. **f**–**g** Anterior caudal (pygal) vertebrae in ventral view. **h**–**i** Incomplete articulated ilium, pubis and ischium in medial and lateral (pubis here partially reconstructed from broken pieces) views, respectively. **j**–**k** Proximal left tibia in caudal view. **l** Tibial diaphysis and distal epiphysis. **m** isolated ungual phalanx in lateral view. **n**–**o** Isolated ungual phalanx in lateral and dorsal views under SEM. ac, acetabulum; apr, anterior process of ilium; bap, basal platform; il, ilium; isc, ischium; lpe, lateral pubic excavation; mc, medial condyle; ns, neural spine; obf, obturator foramen; poz, postzygapophysis; ml, margo lateralis; mv, margo ventralis; prz, prezygapophysis; pu, pubis (incomplete); sy, synapophysis; thf, thyroid fenestra; tp, transverse process; tt, tibial tuberosity; lc, lateral condyle; vk, ventral keel. Scale bars: **a**–**m** = 1 mm; **n**–**p** = 500 µm.

All preserved vertebral centra are procoelic (Fig. 3a–g) and they have a well-developed ventral keel along the midline, creating moderately developed excavations on either side. The centra have a crest connecting the synapophysis to the condyle (margo ventralis) and a dorsally located crest connecting the pre-and postzygapophyses (margo dorsalis). The neural spine is strongly inclined posteriorly and relatively short in lateral view. The synapophyses are circular in cross section, indicating complete fusion of the para- and diapophyses, and are located well anteriorly on the vertebral centrum.

In two articulated mid-dorsal vertebrae (Fig. 3a–d), the synapophysis is positioned higher on the lateral surface of the centra, and it is bound to a conspicuous margo ventralis, which projects ventrocaudally from the synapophysis. The area between the margo ventralis, and the lateral surface of the centrum below the postzygapophysis forms a subtriangular and relatively deep excavation. The prezygapophysis is dorsoventrally thin and directed anterolaterally. It projects farther laterally than the postzygapophysis, which is deeper than the prezygapohysis. No zygosphenes and zygantra are present on any of the observed vertebrae.

Two articulated pygal vertebrae are distinguished by their stout transverse process, robust ventral keel, and centra that are lower and shorter than those of the dorsal vertebrae (Fig. 3f–g). The transverse process is latero-caudodorsally oriented, bearing a slight excavation on its ventroproximal area.

The pelvic girdle (Fig. 3h–i) includes parts of the three right pelvic elements, with the ilium co-ossified to the pubis and ischium, suggesting skeletal maturity of the individual. None of these bones are complete, hampering a full assessment of their anatomy. However, it is possible to identify part of the iliac blade and an anterior process of the ilium. The anteriormost portion of the ilium, forming the anterior pubic process, is impossible to distinguish from the pubic region of the pelvis. The anteromedial surface of the ilium, dorsal to the level of the acetabulum, is shallowly excavated for articulation with the first sacral rib.

The pubis is relatively broad with a large obturator foramen. The pubic symphysis and most of the pubic process are not preserved. The pubis is separated medioventrally from the ischium by the thyroid fenestra. The ischium is missing its posteriormost end, its ventral margin is straight, and the proximal portion is connected to the shaft by the proximal neck.

The preserved left tibia (Fig. 3j–l) has a rounded shaft in cross-section, the proximal epiphysis is triangular, and the distal one is subrectangular. The cnemial crest is expanded forming an expanded tuberosity for the insertion of the *M. quadriceps* (crus extensor). The medial condyle is crested at the posteroproximal region and elevated above the lateral condyle. The latter is medially angled with a flat medial surface for the fibula attachment. The distal epiphysis is marked by a craniocaudal sulcus separating the astragalar condyle from the larger fibular condyle.

Remains of metatarsals have been found, but their exact identification is hampered by incompleteness and disarticulation. Also, the phalanges are mostly incomplete, and their position is uncertain. The unguals bear the expected sickle-like shape, with a median crest on the proximal facet for articulation with distal phalanx (Fig. 3m–o). Interestingly, they also possess a slightly curved ventral plate, which is bifurcated distally and ventrally flattened, forming two basal prongs.

### Comparative anatomy and taxonomy

*Neokotus sanfranciscanus* can be identified as a paramacellodid lizard based on the conspicuous dental morphology of this lineage, characterized by tooth bases that are well expanded labiolingually, whereas the apex remains unexpanded, with the crown apices being lingually concave (Fig. 2). This combination of features is found only in proposed members of Paramacellodidae among all squamates we are aware of, indicating the close affinities between the new species and those traditionally assigned to that family (e.g.^27^). Another feature common to paramacellodids, although not exclusive to them among squamates^28^, is the presence of lingual striations in the tooth crowns. This feature is seemingly present in *Neokotus sanfranciscanus*, but closer inspection via SEM indicates that the putative striated marks may actually represent fragmented parts of the enamel (Fig. 2). As we are not aware of any other descriptions of paramacellodid tooth enamel using SEM imaging, we suggest caution in using this particular feature for taxonomic or phylogenetic purposes as this feature may well be taphonomic or preservational.

Among paramacellodids, *Neokotus sanfranciscanus* is quite distinct from genera such as *Becklesius* and *Atokasaurus*, as well as from *Paramacellodus keebleri*, as those taxa possess chisel shaped tooth apices, usually bearing an accessory lingual cusp. The new taxon is also quite distinct from *Saurillodon*, as the latter has a very robust and dorsoventrally deep dentary. *Neokotus sanfranciscanus* is more similar to other *Paramacellodus* species and also to *Pseudosaurillus*, based on the shape and orientation of its dentition. However, it differs from those genera with regard to the structure of the lower jaw as it lacks the sharp decrease in height of the crista dentalis in the midportion of the dentary when observed in medial view. Also, it has a much more robust ventral crest of the dentary, as well as a splenial that is confined to the posterior half of the dentary. This latter set of features is relatively uncommon among paramacellodids and never observed together in any other taxon (see Table 1). When those features are considered separately, the smoother decline in height of the crista dentalis is also found in *Paramacellodus sinuosus* from the Early Cretaceous of Spain^29^. The more posterior placement of the anterior end of the splenial is also observed in *Saurillus obtusus* from the Early Cretaceous of the UK^27,30^, although in the latter case, it advances further anteriorly, up to the mid-length of the dentary.

Additionally, the midventral crests on the dorsal vertebrae are more strongly developed than in any other paramacellodid vertebra. Importantly, there is no evidence of body osteoderms associated with this specimen, which is also the case with two paramacellodid specimens from the Early Cretaceous of North America^31^. However, given the state of preservation of this material, we consider the current evidence insufficient to call for a definitive absence of body osteoderms on *Neokotus*.

The well-developed cnemial crest, forming an anteriorly projecting expansion, is rarely observed among squamates, but has been previously observed in *Uroplatus*, *Hemidactylus* and within lacertids, and is sometimes referred to as a tibial tuberosity^32^. More importantly, the flat and expanded ventral margin of the ungual phalanges is a feature that is unknown to us in any other squamate, either living or extinct. While it is tempting to infer a unique function for this highly modified ungual morphology, we hesitate to do so in the absence of any functional or ecological correlates amongst modern tetrapods that we are aware of.

### Phylogenetic analyses

**Fig. 4:**
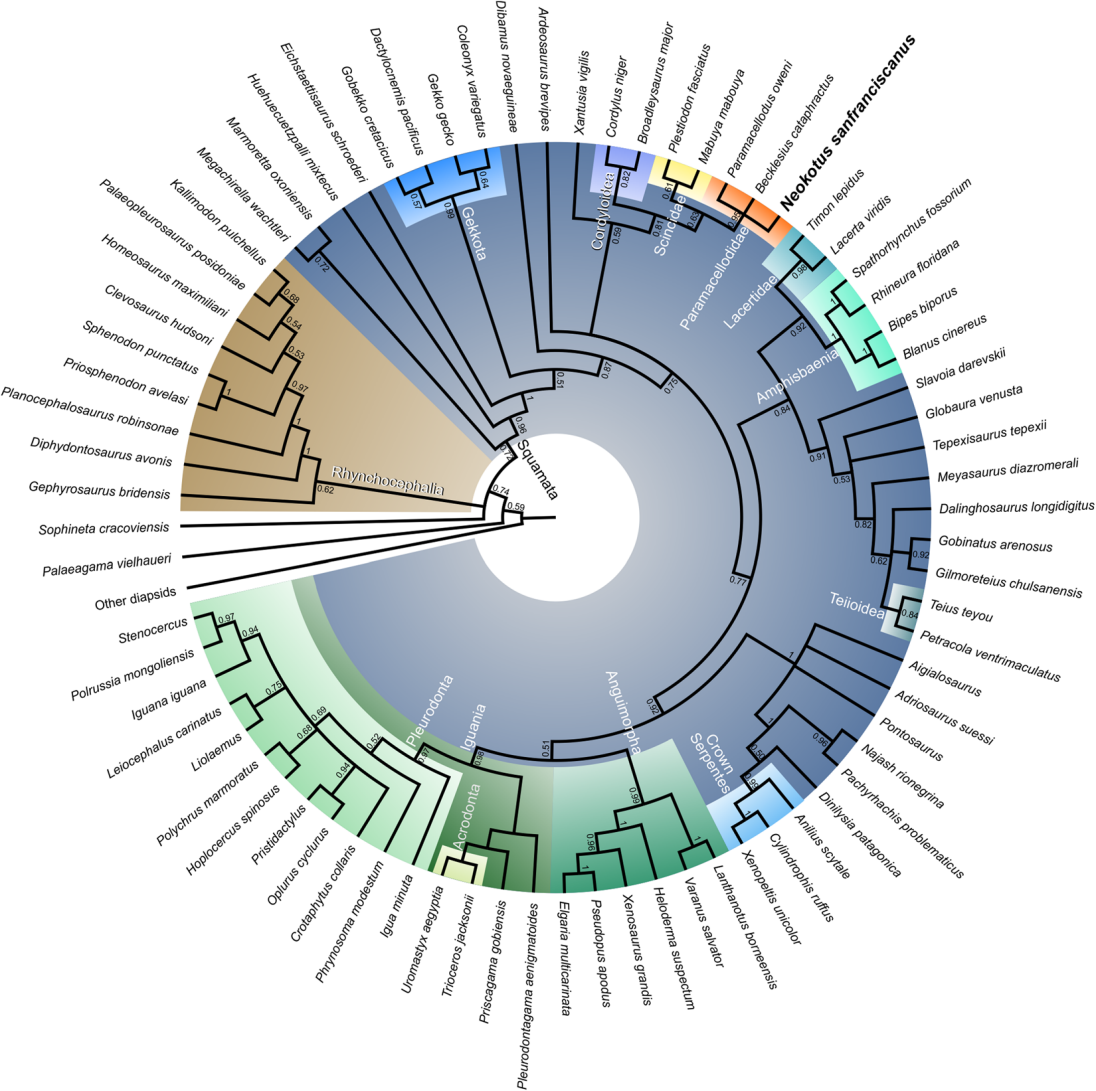
Summarized majority rule consensus tree obtained from the Bayesian inference analysis of the combined morphological and molecular data set. Values at nodes indicate posterior probabilities. Highlighted clades of interest include paramacellodids (orange), scincids (yellow), and cordyloids (purple). For the complete tree see Supplementary Fig. 2.

In all of our analyses we found *Neokotus sanfransiscanus* nested within a monophyletic Paramacellodidae (Fig. 4, Supplementary Figs. 1, 2). As with previous analyses of this data set^14^, and several other studies^31,33–35^, we found paramacellodids to be closely related to scincoids (the clade comprising cordyloids and scincids)[but see^36^ for an exception]. However, relationships between paramacellodids and other scincoids are less well established. Our parsimony analysis found paramacellodids as the sister taxon to cordyloids in agreement with both their original taxonomic placement^27^ and a recent combined evidence Bayesian inference analysis^34^. In contrast, our combined evidence Bayesian inference analysis found paramacellodids to be more closely related to scincids, a relationship also previously recovered by parsimony and Bayesian inference^14^. Other studies, have also found paramacellodids to be the sister taxon to crown scincoids^31,33–35^.

Regardless of the variations in the placement of paramacellodids in relation to cordyloids and scincids, the phylogenetic association between paramacellodids and scincoids makes sense in the light of the fossil record and recent phylogenetic hypotheses. Paramacellodids are among the oldest known groups of squamates recognized in the fossil record—with records dating as far back as the Middle Jurassic^37,38^. Additionally, scincoids have an early diverging position among crown squamates in recent large-scale studies using molecular or combined evidence data [e.g.^14,34,39^], thus suggesting an early diversification of scincoids and closely related fossil lineages (i.e., paramacellodids).

## Discussion

**Fig. 5:**
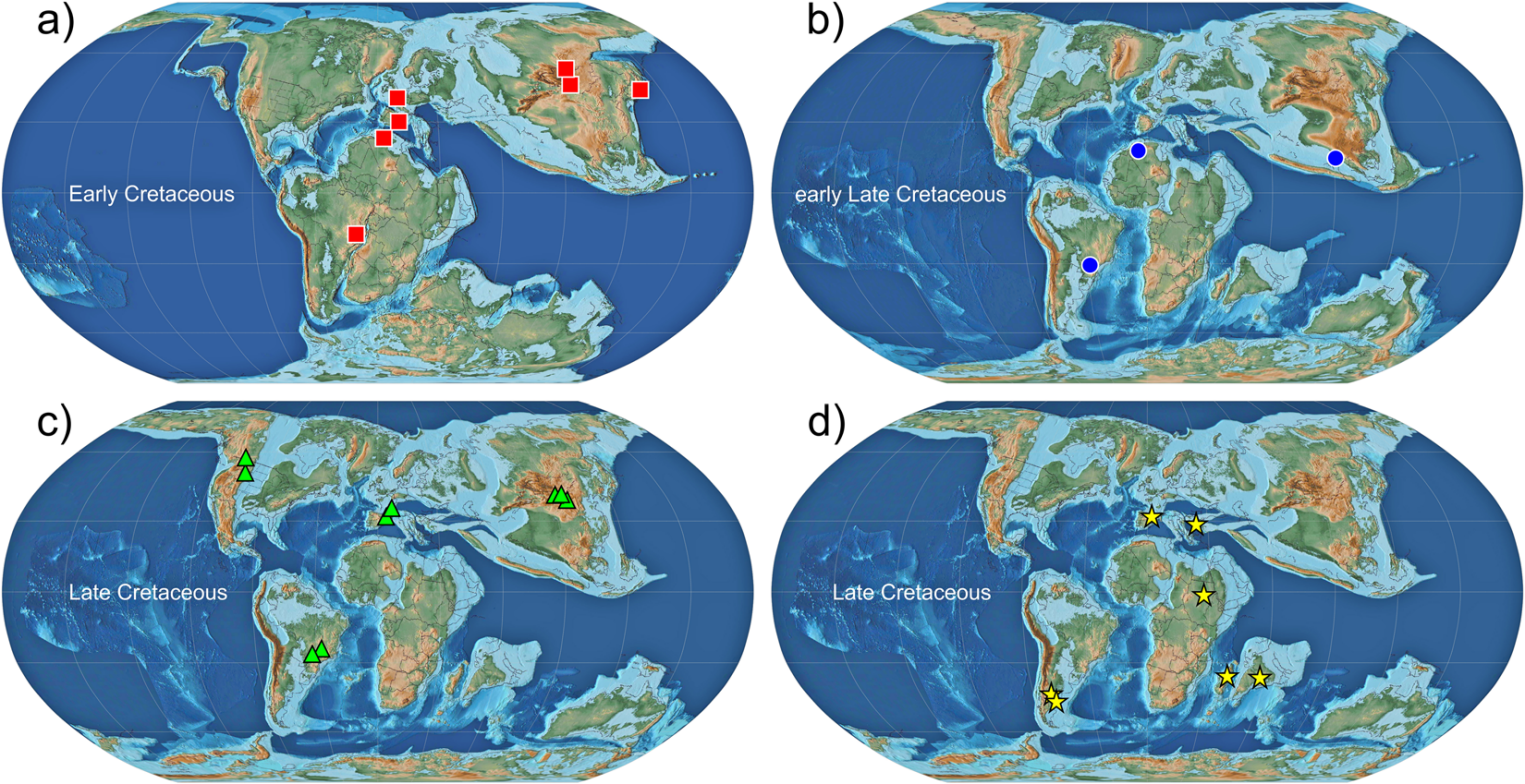
Paleogeographical maps depicting the occurrence of selected clades of Squamata during the Cretaceous. **a** Early Cretaceous (ca. 130 Ma). **b** Early Late Cretaceous (ca. 90 Ma). **c**–**d** Late Cretaceous (ca. 80 Ma). Maps based on Scotese^72^. (squares) Paramacellodidae. (circles) Acrodonta. (triangles) Pleurodonta. (stars) Madtosoiidae. Specimen locations based on Simões et al.^6^, Xing et al.^44^, and additional references in Supplementary Table 1.

Prior to the new data and analyses presented here, the previously oldest South American squamates came from the Crato Formation in northeastern Brazil^4,6^. The age of the Crato Formation is considered to include the entire late Aptian, and possibly encroaching into the early Albian, i.e., ~113–115 Ma^40,41^. The Valanginian age (ca. 133–140 Ma) sediments from where *Neokotus* was recovered are ~20 million years older than the Crato Formation lizards. This pushes back considerably (to the earliest parts of the Cretaceous) the minimum age for the appearance of squamates in South America. Considering that the oldest lizard in Gondwana dates back to the Late Jurassic of Tanzania^42^, that prior to the Aptian/Albian South America was still connected to the African plate (constituting West Gondwana)^43^ (also Fig. 5a), and that the study of South American fossil squamates has only gained some traction in the past decade^12^, it is possible (and perhaps likely) that new discoveries will reveal an even older age for the arrival of squamates in the South American component of West Gondwana.

Previous discoveries have shown that paramacellodids were a quite geographically widespread lizard lineage, occurring in the Late Jurassic of North America, Europe, Africa, Central and East Asia (China)^31,38,44–46^, and in the Early Cretaceous of Europe, Africa, and Central and East Asia (Japan and Mongolia) [e.g.^29,30,47,48^—see also Fig. 5). The new species described here is the first record of paramacellodids in South America, thus expanding the global distribution of this lineage. Previous and new data thus indicate that paramacellodids were the first lineage of squamates to achieve such worldwide distribution (outside the polar circles), with occurrences in quite disparate regions of both Laurasia and Gondwana between the Late Jurassic and Early Cretaceous. Despite our currently limited knowledge of their morphology and taxonomy, given their usually quite fragmentary fossils, paramacellodid lizards have evolved and radiated through an extremely long period of geological time—from the Middle Jurassic to the end of the Cretaceous^49^, a time span of ~100 million years. Those attributes make paramacellodids, as far as the fossil record is concerned, the first successful lineage of globally distributed squamates.

The fossil record of Gondwana has been for a long time a very limited source of data for understanding squamate evolution, both locally and globally, especially concerning terrestrial fully limbed squamates^13^. However, in recent years, a number of studies have provided substantial new information regarding the taxonomic composition of the earliest squamate lineages to radiate into southern continents [e.g.^4,10,50–55^]. These new sources of data concerning both fully limbed squamates and early snakes, along with the new discovery presented herein, indicate some interesting biogeographical patterns (Fig. 5 and Supplementary Information). The early fossil record of squamates in the Cretaceous of South America indicates the presence of both snake and fully limbed squamate lineages that were not exclusive to this continent. Acrodontan and pleurodont iguanians, paramacellodids, and madstoiid snakes are known to have occurred in both South America and Africa during the Cretaceous, also occurring in other geographically disparate regions of both Gondwana and Laurasia (Fig. 5). Such a pattern also extends to aquatic squamates from the Late Cretaceous (mosasaurs)^11^—although the latter is less surprising, giving the greater dispersion capabilities of large bodied marine vertebrates. This indicates that the early squamate fauna of South America, at least at the family level, was well integrated with squamate faunas from other parts of the world. This is in sharp contrast to the squamate diversity in South America from the early Neogene to the present, characterized by many families that are exclusive or almost exclusive to that continent (some later invading Central and North America), such as teiids, gymnophthalmids, different families of pleurodont iguanians (Tropidurinae, Liolaeminae, Leiosaurinae and Hoplocercinae), amphisbaenids, anomalepidids, and micrurids^1,9,56,57^.

It is still difficult to define when squamate faunas in South America became less integrated in taxonomic composition to other areas of the world, and began to develop the endemicity that characterizes its modern fauna^2^. In the Palaeogene, Mesozoic forms that survived the end-Cretaceous mass extinction (e.g., madstoiid snakes) co-existed with more modern looking components of the South American fauna (e.g., boids, tropidophiids, anilioids, teiids, besides pleurodont iguanians that were already present during the Mesozoic)^9,58,59^. By the Neogene, most fossil squamates can be attributed to modern genera, many of which are endemic to South America, such as the teiid *Tupinambis*, the iguanians *Pristidactylus* and *Liolaemus*, and the boid snake *Eunectes*^9^. Therefore, although still limited, the current fossil record suggests that the transition from a cosmopolitan to a more endemic squamate faunas would have occurred during the Cenozoic, most likely along the Paleogene.

This particular pattern has some parallels with the evolution of Cretaceous mammals in South America. At one point it was not known whether the South American mammalian fauna during the Cretaceous was endemic to that continent or if it was part of a more widespread distribution of taxa. More recently, fossil discoveries in Madagascar, continental Africa, India, and other parts of Gondwana revealed that at least some groups of South American Mesozoic mammals were distributed throughout Gondwana^60,61^. Later in the Cenozoic, the geographic isolation of South America contributed to the evolution of an unique endemic fauna of placental and marsupial mammals, including ameridelphian marsupials, xenarthrans, litopterns, and notoungulates^62,63^. The long period of South American isolation from other continents during the Cenozoic is likely to have contributed to the development of an equally more endemic squamate fauna, in a similar manner to what happened to its mammalian faunas during the last 66 million years.

Despite receiving much less attention and having much fewer data points compared to their fossil record in the northern hemisphere, early evolving squamates from Gondwana have provided answers to key-questions regarding the origin and early biogeography of important squamates lineages—e.g.^9,10,50,64,65^. Our findings herein demonstrate that the appearance of squamates in South America is much older than previously assumed. Additionally, the widespread geographic distribution of Gondwanan (including South American) squamate families during the Cretaceous suggests those faunas were not yet specialized to clearly distinct biogeographical regions, in contrast to what is observed during the Cenozoic. This could mean either that dispersion routes between continents were still operating (e.g., dispersion between South America and Africa or Antarctica was still possible, at least intermittently, until the end of the Cretaceous), or that those globally distributed faunal components were remnants of more ancient worldwide distributions. At least in the case of paramacellodids, their extremely old fossil record and phylogenetically inferred divergence times^14,35^—back in the Jurassic—suggests the latter factor might be of greater significance to explain their distribution patterns. South American squamate fossils have been and continue to be very important to not only understand the deep time origins of Gondwanan squamates, but also to fully understand global patterns of early squamate biogeographic history.

## Methods

### Anatomical nomenclature

Throughout the text, we follow previously used anatomical nomenclature^66,67^.

### Photography and scanning electron microscope (SEM) imaging

The specimen described here was observed under a Leica DM750 dissecting stereomicroscope and photographed using a AmScope FMA 050 camera. High-resolution images of dental structures and unguals were obtained by coating the specimen with carbon and using a scanning electron microscope JEOL JSM-6510 in the Instituto de Geociências, at Universidade Federal de Minas Gerais.

### Morphological and molecular data sets

In order to assess the phylogenetic placement of *Neokotus sanfranciscanus*, we utilized the previously published morphological and molecular data sets of Simões et al.^14^. We included the new taxon and another well-preserved paramacellodid species, *Becklesius cataphractus* [Early Cretaceous of Spain^29^], in the aforementioned data set, which already included one paramacellodid (*Paramacellodus oweni*, Early Cretaceous of the UK and possibly USA). The updated data set^14^ with those additions is available as online Supplementary Data in the journal’s website. The vast majority of paramacellodid specimens described so far are composed of isolated fragments, few of which can be assigned to a particular species. The paramacellodid taxa included in our analyses are three of the most complete and phylogenetically informative taxa currently available; most other taxa are represented by very fragmentary or isolated materials. These OTUs also represent geographically and morphologically disparate representatives of this lineage, which are rarely included in broad-scale assessments of squamate relationships.

Additionally, in the original version of this data set, no characters were included to assess the monophyly of paramacellodids, as only one member of that group was part of the study. We therefore included two additional characters that can be assessed across most paramacellodid species as well as other squamates (with low levels of missing data) and are considered to be typical features of this lineage:^27^ Character 348. Dentition, tooth shape, labiolingual expansion: absent (0)/expanded at tooth base only (1)/expanded from base to apex (2) (NEW). 349. Dentition, crown apices, lingually concave: absent (0)/present (1) (NEW). In this way, we can test if those features indeed represent paramacellodid synapomorphies when balanced against several other morphological characters in terms of overall character agreement within the analysis.

### Parsimony analysis

Analyses were conducted in TNT v. 1.1^68^ using the New Technology Search (NTS) algorithms. Tree searches were conducted using 1000 initial trees by random addition sequences (RAS) with 100 iterations/round for each of the four NTS algorithms: Sectorial Search, Ratchet, Drift and Tree Fusing. The output trees were used as the starting trees for subsequent runs, using 1000 iterations/rounds of each of the NTS algorithms. The latter step was repeated once again, and the final output trees were filtered for all the most parsimonious trees (MPTs). A total of 495 MPTs were obtained with 2279 steps each.

### Bayesian inference analyses

Analyses were conducted using Mr. Bayes v. 3.2.6^69^ on the Cedar computer cluster, made available through Compute Canada. As there were no changes to the molecular data set we used^14^, molecular partitions and models of evolution were the same as in that study. The morphological partition was analyzed with the Mkv model. Convergence of independent runs was assessed using: average standard deviation of split frequencies (ASDSF ~ 0.01), potential scale reduction factors (PSRF ≈ 1 for all parameters), and effective sample size (ESS) for each parameter was >200.

### Nomenclatural acts

This published work and the nomenclatural acts it contains have been registered in ZooBank, the proposed online registration system for the International Code of Zoological Nomenclature (ICZN). The ZooBank LSIDs (Life Science Identifiers) can be resolved and the associated information viewed through any standard web browser by appending the LSID to the prefix “http://zoobank.org/“. The LSIDs for this publication are: urn:lsid:zoobank.org:pub:B9CE4931-857A-4886-811D-D0A7D574FD27; urn:lsid:zoobank.org:act:AF9D0A67-4C52-44D7-B9EA-97F3231B8209; urn:lsid:zoobank.org:act:68BF448E-D95A-45C9-8BA7-B7982C5F4558.

### Reporting summary

Further information on research design is available in the Nature Research Reporting Summary linked to this article.

## Supplementary information

Supplementary Information

Supplementary Data 1

Supplementary Data 2

Supplementary Data 3

Reporting Summary

Description of Additional Supplementary Files

## Data Availability

The datasets analysed during the current study are included in this published article (and its Supplementary Information files).
