# Supplementary material for: Discovery of the oldest South American fossil lizard illustrates the cosmopolitanism of early South American squamates
Source: Commun Biol. 2020 Apr 29;3:201. doi: 10.1038/s42003-020-0926-0 (PMC7190622; doi:10.1038/s42003-020-0926-0)
Supplement: Supplementary file 1 — Supplementary Information [file 42003_2020_926_MOESM1_ESM.pdf]

Supplementary Information

The oldest fossil lizard from South America and the cosmopolitanism of early South American squamates

1. Supplementary Figures

2. Supplementary Table

3. Supplementary References

1. Supplementary Figures

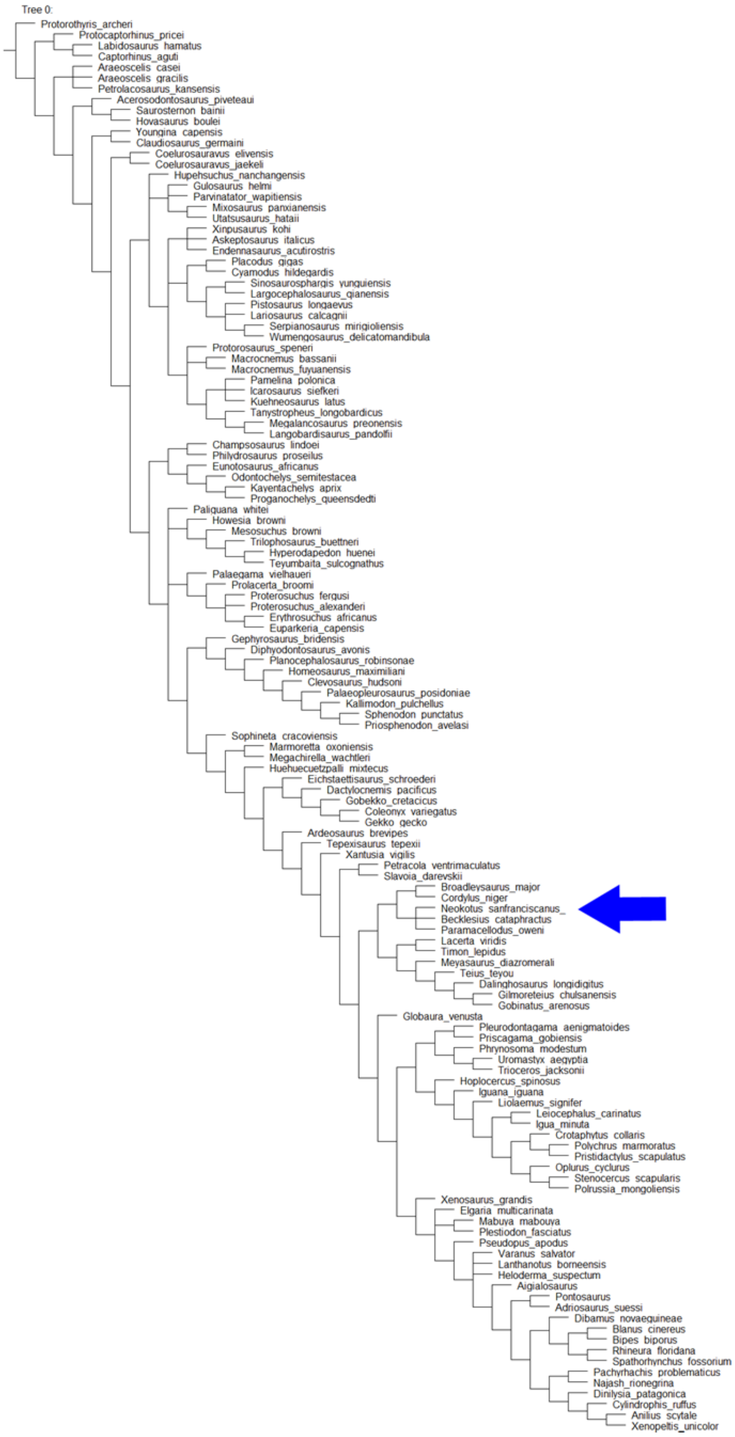

Supplementary Figure 1. Strict consensus tree obtained from 495 most parsimonious trees (2,279 steps each; CI = 0.18; RI = 64) from the analyses of morphological data only using maximum parsimony.

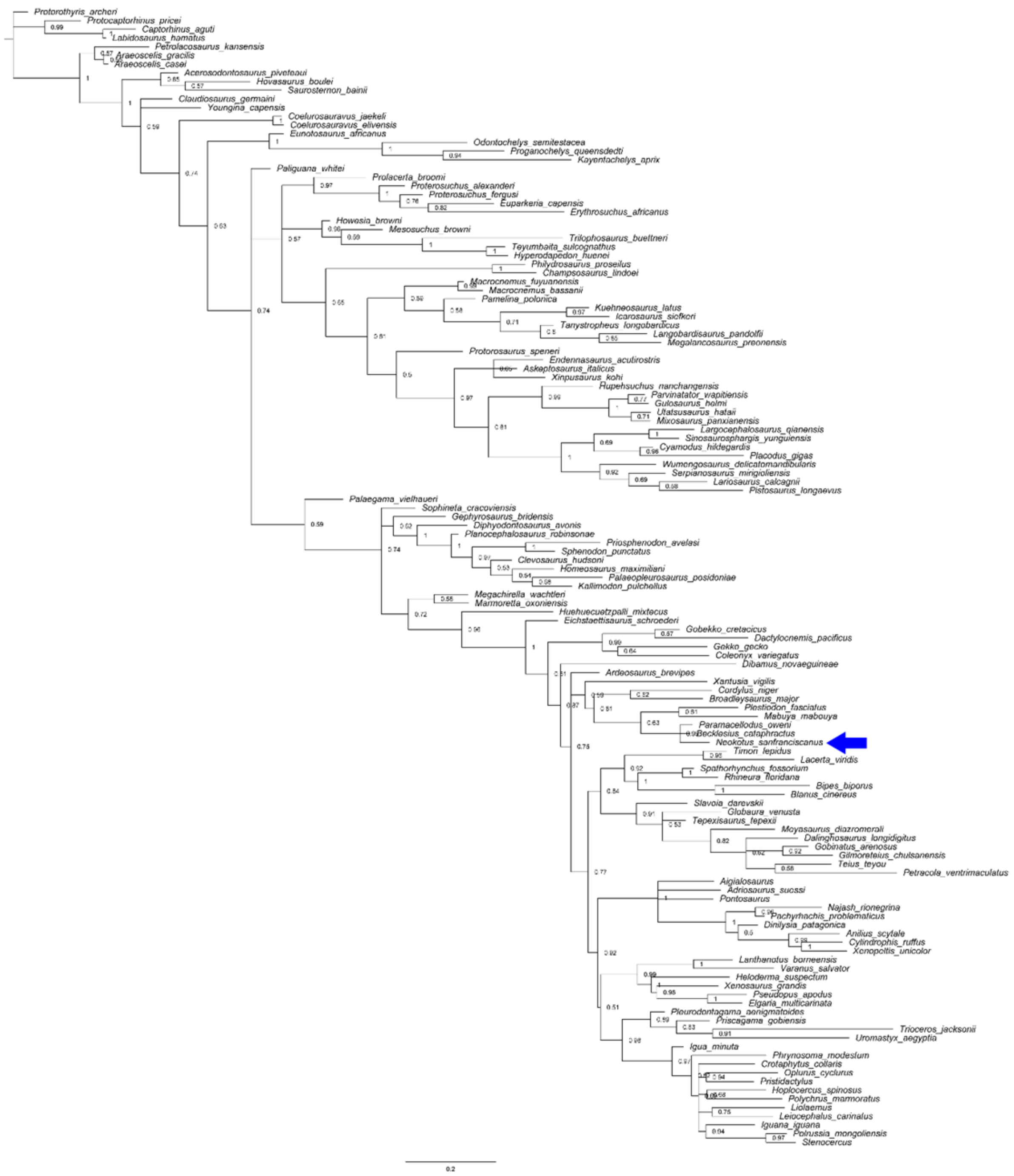

Supplementary Figure 2. Majority rule consensus tree obtained from the Bayesian inference analyses of the combined morphological and molecular data. Node values indicate posterior probabilities.

2. Supplementary Table

Supplementary Table 1. Reference data for individual taxon occurrences provided in figure 5.

	Taxon	Provenance	Age	Ref.
**Paramacellodidae**	*Paramacellodus oweni* Hoffstetter, 1967 (= *Saurillus robustensis* Hoffstetter, 1967; *Becklesiosaurus scincoides* Hoffstetter, 1967)	United Kingdom	Berriasian	(Hoffstetter, 1967)
	Paramacellodus marocensis Richter, 1994	Morocco	?Berriasian	(Richter, 1994)
	Paramacellodidae indet.	Japan	Early Cretaceous	(Evans *et al.*, 1998)
	Paramacellodus sinuosus Richter, 1994	Spain	Barremian	(Richter, 1994)
	cf. *Paramacellodus*	Russia	Barremian/Aptian	(Averianov and Skutchas, 1999)
	Becklesius cataphractus Richter, 1994	Spain	Barremian	(Richter, 1994)
	Paramacellodidae indet.	Mongolia	Aptian/Albian	(Evans and Chure, 1998)
**Acrodonta**	*Gueragama sulamericana* Simões et al., 2015	Brazil	Turonian-Campanian	(Simões *et al.*, 2015)
	Jeddaherdan aleadonta Apesteguía et al., 2016	Morocco	Cenomanian	(Apesteguía *et al.*, 2016)
	Stem chameleons and agamids in amber: chamaeleonid re-interpreted as an albanerpetonid amphibian (Matsumoto and Evans, 2018)	Myanmar	Albian-Cenomanian	(Daza *et al.*, 2016; Matsumoto and Evans, 2018)
	Not included: *Xianglong zhaoi*: Non-diagnostic material—(Evans and Manabe, 2009) and this work	China	Aptian/Albian	(Li *et al.*, 2007; Evans and Manabe, 2009)
**Pleurodonta**	*Pristiguana brasiliensis* Estes & Price, 1973	Brazil	Maastrichtian	(Estes and Price, 1973)
	Brasiliguana prudentis Nava & Martinelli, 2011	Brazil	Turonian-Santonian	(Nava and Martinelli, 2011)
	Iguanid	Argentina	Cenomanian-Turonian	(Apesteguía *et al.*, 2005)
	Cnephasaurus locustivorus Gao & Fox, 1996	USA	Late Cretaceous	(Gao and Fox, 1996; Nydam, 2013)
	Indet.	USA	Late Cretaceous	(Gao and Fox, 1996; Nydam, 2013)
	Pariguana lancensis Longrich et al., 2012	USA	Maastrichtian?	(Longrich *et al.*, 2012)
	Iguanian	USA	Campanian	(DeMar *et al.*, 2017)
	Possible Iguanians	Spain	Maastrichtian	(Astibia *et al.*, 1990; Rage, 1999; Blain *et al.*, 2010; Blanco *et al.*, 2016)
	Iguanian?	France	Late Cretaceous	(Sigé *et al.*, 1997)
	Isodontosaurus gracilis Gilmore, 1943	Mongolia	Middle Campanian	(Gao and Norell, 2000)
	Saichangurvel davidsoni Conrad & Norell, 2007	Mongolia	Middle Campanian	(Conrad and Norell, 2007)
	Temujinia ellisoni Gao & Norell, 2000	Mongolia	Middle Campanian	(Gao and Norell, 2000)
	Zapsosaurus sceliphros Gao & Norell, 2000	Mongolia	Middle	(Gao and Norell,

			Campanian	2000)
	Ctenomastax parva Gao & Norell, 2000	Mongolia	Middle Campanian	(Gao and Norell, 2000)
	Polrussia mongoliensis Borsuk-Bialynicka & Alifanov, 1991	Mongolia	Late Campanian	(Gao and Norell, 2000)
	Igua minuta Borsuk-Bialynicka & Alifanov, 1991	Mongolia	Late Campanian	(Borsuk-Białynicka and Alifanov, 1991)
	Desertiguana gobiensis Alifanov, 2013	Mongolia	Late Campanian	(Alifanov, 2013)
	Anchaurosaurus gilmorei Gao & Hou, 1995	China	Middle Campanian	(Gao and Hou, 1995; Gao and Hou, 1996)
	Xihaina aquilonia Gao & Hou, 1995	China	Middle Campanian	(Gao and Hou, 1995; Gao and Hou, 1996)
**Madtsoiidae**	*Dinilysia patagonica* Woodward, 1901	Argentina	Coniacian	(Woodward, 1901; Caldwell and Albino, 2001)
	Najash rionegrina Apesteguía & Zaher, 2006	Argentina	Cenomanian-Turonian	(Apesteguía and Zaher, 2006)
	Rionegrophis madtsoioides Albino, 1986	Argentina	Campanian-early Maastrichtian	(Albino, 1986)
	Alamitophis argentinus Albino, 1986	Argentina	Campanian-early Maastrichtian	(Albino, 1986)
	Alamitophis elongatus Albino, 1994	Argentina	Campanian-early Maastrichtian	(Albino, 1986; Albino, 1994)
	Patagoniophis parvus Albino, 1986	Argentina	Campanian-early Maastrichtian	(Albino, 1986)
	Madtsoidae indet.	Sudan	Cenomanian	(Rage and Werner, 1999)
	Madtsoia madagascariensis Hoffstetter, 1961	Madagascar	Santonian or Campanian	(Hoffstetter, 1961)
	Madtsoia pisdurensis Mohabey et al., 2011	India	Maastrichtian	(Mohabey *et al.*, 2011)
	Madtsoia laurasiae Rage, 1996	Romania	Maastrichtian	(Astibia *et al.*, 1990; Rage, 1996)
	Nidophis insularis Vasile et al., 2013	Romania	Maastrichtian	(Vasile *et al.*, 2013)
	Madtsoiidae indet.	Romania	Maastrichtian	(Folie and Codrea, 2005; Vasile *et al.*, 2013)

3. Supplementary References

Albino, A. M. 1986. Nuevos boidae madtsoiinae en el cretácico tardio de patagonia (formación los alamitos, río negro, argentina). IV Congreso Argentino de Paleontología y Bioestratigrafía, Mendoza.

Albino, A. M. 1994. Una nueva serpiente (reptilia) en el cretácico superior de patagonia, argentina. Pesq. Geociênc. 21, 58-63.

Alifanov, V. R. 2013. *Desertiguana gobiensis* gen. Et sp. Nov., a new lizard (phrynosomatidae, iguanomorpha) from the upper cretaceous of mongolia. Paleontol. J. 47, 417-424.

Apesteguía, S., Agnolín, F. L., Lio, G. L. 2005. An early late cretaceous lizard from patagonia, argentina. C. R. Paleoevol. 4, 311-315.

Apesteguía, S., Daza, J. D., Simões, T. R., Rage, J. C. 2016. The first iguanian lizard from the mesozoic of africa. Royal Soc. Open Sci. 3, 160462.

Apesteguía, S., Zaher, H. 2006. A cretaceous terrestrial snake with robust hindlimbs and a sacrum. Nature 440, 1037-1040.

Astibia, H., Buffetaut, E., Buscalioni, A. D., Cappetta, H., Corral, C., Estes, R., Garcia-Garmilla, F., Jaeger, J. J., Jimenez-Fuentes, E., Loeuff, J. L., Mazin, J. M., Orue-Etxebarria, X., Pereda-Suberbiola, J., Powell, J. E., Rage, J. C., Rodriguez-Lazaro, J., Sanz, J. L., Tong, H. 1990. The fossil vertebrates from lano (basque country, spain); new evidence on the composition and affinities of the late cretaceous continental faunas of europe. Terra Nova 2, 460-466.

Averianov, A. O., Skutchas, P. P. 1999. Paramacellodid lizard (squamata, scincomorpha) from the early cretaceous of transbaikalia. Russ. J. Herpetol. 6, 115-117.

Blain, H.-A., Canudo, J.-I., Cuenca-Bescós, G., López-Martínez, N. 2010. Amphibians and squamate reptiles from the latest maastrichtian (upper cretaceous) of blasi 2 (huesca, spain). Cretac. Res. 31, 433-446.

Blanco, A., Bolet, A., Blain, H.-A., Fondevilla, V., Marmi, J. 2016. Late cretaceous (maastrichtian) amphibians and squamates from northeastern iberia. Cretaceous Res. 57, 624-638.

Borsuk-Białynicka, M., Alifanov, V. R. 1991. First asiatic 'iguanid' lizards in the late cretaceous of mongolia. Acta Palaeontol. Pol. 36, 325-342.

Caldwell, M. W., Albino, A. M. 2001. Palaeoenvironment and palaeoecology of three cretaceous snakes: *Pachyophis*, *pachyrhachis*, and *dinilysia*. Acta Palaeontol. Pol. 46, 203-218.

Conrad, J. L., Norell, M. A. 2007. A complete late cretaceous iguanian (squamata, reptilia) from the gobi and identification of a new iguanian clade. Am. Mus. Novit. 3584, 1 - 47.

Daza, J. D., Stanley, E. L., Wagner, P., Bauer, A. M., Grimaldi, D. A. 2016. Mid-cretaceous amber fossils illuminate the past diversity of tropical lizards. Science advances 2, e1501080.

DeMar, D. G., Conrad, J. L., Head, J. J., Varricchio, D. J., Wilson, G. P. 2017. A new late cretaceous iguanomorph from north america and the origin of new world pleurodonta (squamata, iguania). Proc. R. Soc. Lond. B. Biol. Sci. 284, 20161902.

Estes, R., Price, L. I. P. 1973. Iguanid lizard from the upper cretaceous of brazil. Science 180, 784–751.

Evans, S. E., Chure, D. C. 1998. Paramacellodid lizard skulls from the jurassic morrison formation at dinosaur national monument, utah. J. Vert. Paleontol. 18, 99-114.

Evans, S. E., Manabe, M. 2009. The early cretaceous lizards of eastern asia: New material of sakurasaurus from japan. Spec. Pap. Palaeontol. 81, 43-59.

Evans, S. E., Manabe, M., Cook, E., Hirayama, R., Isaji, S., Nicholas, C., Unwin, D., Yabumoto, Y. 1998. An early cretaceous small vertebrate assemblage from gifu prefecture, japan. Bulletin of the New Mexico Museum of Natural History and Science 14, 183-186.

Folie, A., Codrea, V. 2005. New lissamphibians and squamates from the maastrichtian of haţeg basin, romania. Acta Palaeontol. Pol. 50, 57-71.

Gao, K.-Q., Fox, R. C. 1996. Taxonomy and evolution of late cretaceous lizards (reptilia:Squamata) from western canada. Bull. Carnegie Mus. Nat. Hist. 33, 1-107.

Gao, K.-Q., Hou, L. 1995. Iguanians from the upper cretaceous djadochta formation, gobi desert, china. J. Vert. Paleontol. 15, 57-78.

Gao, K.-Q., Hou, L. 1996. Systematics and taxonomic diversity of squamates from the upper cretaceous djadochta formation, bayan mandahu, gobi desert, people's republic of china. Can. J. Earth Sci. 33, 578-598.

Gao, K.-Q., Norell, M. A. 2000. Taxonomic composition and systematics of late cretaceous lizard assemblages from ukhaa tolgod and adjacent localities, mongolian gobi desert. Bull. Am. Mus. Nat. Hist. 249, 1-118.

Hoffstetter, R. 1961. Nouveaux restes d'un serpent boïdé (*madtsoia madagascariensis* nov. Sp.) dans le crétacé supérieur de madagascar. Bull. Mus. Nat. d'Hist. Nat. 33, 152–160.

Hoffstetter, R. 1967. Coup d'oeil sur le sauriens (= lacertiliens) des couches de purbeck (jurasique supérieur d'angleterre) - résumé d'un mémoire. Colloq. Int. CNRS 163, 349–371.

Li, P.-P., Gao, K.-Q., Hou, L.-H., Xu, X. 2007. A gliding lizard from the early cretaceous of china. Proc. Natl. Acad. Sci. USA 104, 5507-5509.

Longrich, N. R., Bhullar, B.-A. S., Gauthier, J. A. 2012. Mass extinction of lizards and snakes at the cretaceous–paleogene boundary. PNAS 109, 21396-21401.

Matsumoto, R., Evans, S. E. 2018. The first record of albanerpetontid amphibians (amphibia: Albanerpetontidae) from east asia. PLOS ONE 13, e0189767.

Mohabey, D. M., Head, J. J., Wilson, J. A. 2011. A new species of the snake madtsoia from the upper cretaceous of india and its paleobiogeographic implications. J. Vert. Paleontol. 31, 588-595.

Nava, W. R., Martinelli, A. G. 2011. A new squamate lizard from the upper cretaceous adamantina formation (bauru group), são paulo state, brazil. An. Acad. Bras. Cienc. 83, 291-299.

Nydam, R. L. 2013. Squamates from the jurassic and cretaceous of north america. Palaeobio Palaeoenv 93, 535-565.

Rage, J.-C. 1996. Les madtsoiidae (reptilia, serpentes) du crétacé supérieur d'europe: Témoins gondwaniens d'une dispersion transtéthysienne. C. R. Acad. Sci. S. IIa 322, 603–608.

Rage, J., Werner, C. 1999. Mid-cretaceous (cenomanian) snakes from wadi abu hashim, sudan: The earliest snake assemblage. Palaeontol. Afr. 35, 85-110.

Rage, J. C. 1999. Squamates (reptilia, squamata) from the upper cretaceous of laño (basque country, spain). Estudios del Museo de Ciencias Naturales de Álava 14, 121-133.

Richter, A. 1994. Lacertilia aus der unteren kreide von uña und galve (spanien) und anoual (marokko). Berliner Geowiss Abh Reihe E (Paläebiol) 14, 1 – 147.

Sigé, B., Buscalioni, A. D., Duffaud, S., Gayet, M., Orth, B., Rage, J.-C., Sanz, J. L. 1997. Etat des données sur le gisement crétacé supérieur continental de champ-garimond (gard, sud de la france). Münchner Geowiss. Abh. 34A, 111-130.

Simões, T. R., Wilner, E., Caldwell, M. W., Weinschütz, L. C., Kellner, A. W. A. 2015. A stem acrodontan lizard in the cretaceous of brazil revises early lizard evolution in gondwana. Nat. Comm. 6, 9149.

Vasile, Ş., Csiki-Sava, Z., Venczel, M. 2013. A new madtsoiid snake from the upper cretaceous of the hateg basin, western romania. J. Vert. Paleontol. 33, 1100-1119.

Woodward, A. S. 1901. On some extinct reptiles from patagonia, of the genera *miolania*, *dinilysia*, and *genyodectes*. Proc. Zool. Soc. Lond. 70, 169-184.
