# Supplementary material for: Discovery of the oldest South American fossil lizard illustrates the cosmopolitanism of early South American squamates
Source: Commun Biol. 2020 Apr 29;3:201. doi: 10.1038/s42003-020-0926-0 (PMC7190622; doi:10.1038/s42003-020-0926-0)
Supplement: Supplementary file 5 — Reporting Summary [file 42003_2020_926_MOESM5_ESM.pdf]

nature research

Corresponding author(s): Jonathas S. Bittencourt

Last updated by author(s): Mar 24, 2020

Reporting Summary

Nature Research wishes to improve the reproducibility of the work that we publish. This form provides structure for consistency and transparency in reporting. For further information on Nature Research policies, see Authors & Referees and the Editorial Policy Checklist.

Statistics

For all statistical analyses, confirm that the following items are present in the figure legend, table legend, main text, or Methods section.

n/a	Confirmed	
☒	☐	The exact sample size (*n*) for each experimental group/condition, given as a discrete number and unit of measurement
☒	☐	A statement on whether measurements were taken from distinct samples or whether the same sample was measured repeatedly
☒	☐	The statistical test(s) used AND whether they are one- or two-sided<br>*Only common tests should be described solely by name; describe more complex techniques in the Methods section.*
☒	☐	A description of all covariates tested
☒	☐	A description of any assumptions or corrections, such as tests of normality and adjustment for multiple comparisons
☒	☐	A full description of the statistical parameters including central tendency (e.g. means) or other basic estimates (e.g. regression coefficient) AND variation (e.g. standard deviation) or associated estimates of uncertainty (e.g. confidence intervals)
☒	☐	For null hypothesis testing, the test statistic (e.g. *F*, *t*, *r*) with confidence intervals, effect sizes, degrees of freedom and *P* value noted<br>*Give P values as exact values whenever suitable.*
☐	☒	For Bayesian analysis, information on the choice of priors and Markov chain Monte Carlo settings
☒	☐	For hierarchical and complex designs, identification of the appropriate level for tests and full reporting of outcomes
☒	☐	Estimates of effect sizes (e.g. Cohen's *d*, Pearson's *r*), indicating how they were calculated

Our web collection on statistics for biologists contains articles on many of the points above.

Software and code

Policy information about availability of computer code

Data collection	No software was used for data collection.
Data analysis	Our data was compiled in Mesquite (v. 3.04); molecular alignments were performed with MAFFT (Multiple Sequence Alignment Software Version 7); our analysis were performed in the phylogenetic software Mr, Bayes (v. 3.2.6) and BEAST2.

For manuscripts utilizing custom algorithms or software that are central to the research but not yet described in published literature, software must be made available to editors/reviewers. We strongly encourage code deposition in a community repository (e.g. GitHub). See the Nature Research guidelines for submitting code & software for further information.

Data

Policy information about availability of data

All manuscripts must include a data availability statement. This statement should provide the following information, where applicable:

- Accession codes, unique identifiers, or web links for publicly available datasets
- A list of figures that have associated raw data
- A description of any restrictions on data availability

The datasets analysed during the current study are included in this published article (and its supplementary information files).

Field-specific reporting

Please select the one below that is the best fit for your research. If you are not sure, read the appropriate sections before making your selection.

☐ Life sciences ☐ Behavioural & social sciences ☒ Ecological, evolutionary & environmental sciences

Ecological, evolutionary & environmental sciences study design

All studies must disclose on these points even when the disclosure is negative.

Study description	We studied the anatomy of a new lizard species from the Cretaceous of southeast Brazil and investigate its phylogentic relationships by means of parsimony and Bayesian analyses on morphological (personally collected in museum collections) and molecular (available online) data from all major lineages of diapsid reptiles.
Research sample	Other than the specimen originally described in the paper, the collected data from 131 species of extant and extinct reptiles from over 50 different museum collections across the world, spanning in geological time from 307 myr to the present.
Sampling strategy	Our samples were obtained in a way to maximize the representation of the modern diversity of reptiles, as well as the representation of fossil lineages, focusing on the earliest and most complete fossils from each reptile clades studied here.
Data collection	Data was collected using digital photography and CT scanning technologies for the morphological data, whereas molecular data was obtained from GenBank.
Timing and spatial scale	N/A
Data exclusions	No data was excluded from the analyses.
Reproducibility	Some of the analyses conducted were repeated multiple times, yielding similar results in terms of posterior parameter distributions.
Randomization	*Describe how samples/organisms/participants were allocated into groups. If allocation was not random, describe how covariates were controlled. If this is not relevant to your study, explain why.*
Blinding	Blinding was not relevant to this data set, and set of analyses.

Did the study involve field work? ☒ Yes ☐ No

Field work, collection and transport

Field conditions	The field work was conducted during 2017 winter. No environmental condition is relevant here.
Location	The new material described in the paper was collected in João Pinheiro, north Minas Gerais State, southeast Brazil.
Access and import/export	The collection complied with the current Brazilian legislation for fossil extraction.
Disturbance	No disturbance has been caused by the sample extraction.

Reporting for specific materials, systems and methods

We require information from authors about some types of materials, experimental systems and methods used in many studies. Here, indicate whether each material, system or method listed is relevant to your study. If you are not sure if a list item applies to your research, read the appropriate section before selecting a response.

Materials & experimental systems

n/a	Involved in the study	
☒	☐	Antibodies
☒	☐	Eukaryotic cell lines
☐	☒	Palaeontology
☒	☐	Animals and other organisms
☒	☐	Human research participants
☒	☐	Clinical data

Methods

n/a	Involved in the study	
☒	☐	ChIP-seq
☒	☐	Flow cytometry
☒	☐	MRI-based neuroimaging

Palaeontology

Specimen provenance	The new material described in the paper was collected in João Pinheiro, north Minas Gerais State, southeast Brazil. The Brazilian legislation, including the Federal Law-Decree 4146, published on March 4, 1941, confers automatic permit to all Brazilian universities, to one of which the corresponding author is filiated, to perform fossil collection in Brazil's territory.
Specimen deposition	The specimen is deposited in the Institute of Geosciences of the Federal University of Minas Gerais (IGC-P 0085) and will become available for public consulting as soon as the paper is published.

Dating methods

No new dates were provided. We followed a previous work which dated the fossil-bearing stratigraphic layer by means of microfossils' ranges (ostracods). The referenced work is explicitly cited within the manuscript.

☐ Tick this box to confirm that the raw and calibrated dates are available in the paper or in Supplementary Information.
