# Supplementary material for: Discovery of the oldest South American fossil lizard illustrates the cosmopolitanism of early South American squamates
Source: Commun Biol. 2020 Apr 29;3:201. doi: 10.1038/s42003-020-0926-0 (PMC7190622; doi:10.1038/s42003-020-0926-0)
Supplement: Supplementary file 6 — Description of Additional Supplementary Files [file 42003_2020_926_MOESM6_ESM.pdf]

## Description of Additional Supplementary Files

**File Name**: **Supplementary Data 1**
**Description**: Morphological phylogenetic data matrix in Nexus format.

**File Name**: **Supplementary Data 2**
**Description**: Morphological data matrix plus Mr. Bayes block containing all necessary coding for morphology only analysis in Nexus format

**File Name**: **Supplementary Data 3**
**Description**: Morphological and molecular data matrix plus Mr. Bayes block containing all necessary coding for combined evidence analyses in Nexus format.
